# Single-Nucleotide Polymorphisms in Oxidative Stress-Related Genes and the Risk of a Stroke in a Polish Population—A Preliminary Study

**DOI:** 10.3390/brainsci11030391

**Published:** 2021-03-19

**Authors:** Ewelina Synowiec, Paulina Wigner, Natalia Cichon, Cezary Watala, Piotr Czarny, Joanna Saluk-Bijak, Elzbieta Miller, Tomasz Sliwinski, Ewa Zielinska-Nowak, Michal Bijak

**Affiliations:** 1 Laboratory of Medical Genetics, Faculty of Biology and Environmental Protection, University of Lodz, Pomorska 141/143, 90-236 Lodz, Poland; ewelina.synowiec@biol.uni.lodz.pl (E.S.); paulina.wigner@biol.uni.lodz.pl (P.W.); 2 Biohazard Prevention Center, Faculty of Biology and Environmental Protection, University of Lodz, Pomorska 141/143, 90-236 Lodz, Poland; natalia.cichon@biol.uni.lodz.pl (N.C.); michal.bijak@biol.uni.lodz.pl (M.B.); 3 Department of Haemostatic Disorders, Chair of Biomedical Sciences, Medical University of Lodz, Mazowiecka 6/8, 92-215 Lodz, Poland; cezary.watala@umed.lodz.pl; 4 Department of Medical Biochemistry, Medical University of Lodz, Mazowiecka 6/8, 92-215 Lodz, Poland; piotr.czarny@umed.lodz.pl; 5 Department of General Biochemistry, Faculty of Biology and Environmental Protection, University of Lodz, Pomorska 141/143, 90-236 Lodz, Poland; joanna.saluk@biol.uni.lodz.pl; 6 Department of Neurological Rehabilitation, Medical University of Lodz, Milionowa 14, 93-113 Lodz, Poland; elzbieta.dorota.miller@umed.lodz.pl (E.M.); ewa.zielinska@umed.lodz.pl (E.Z.-N.)

**Keywords:** stroke, genetic variation, oxidative-stress, SNP, single nucleotide polymorphism, ischemic stroke

## Abstract

The present preliminary case-control study was undertaken to detect the potential association of six single nucleotide polymorphisms (SNPs) in oxidative stress-related genes: *SOD2* (c.47T > C; rs4880), *CAT* (c.-89A > T; rs7943316), *GPX4* (c.660T > A; rs713041), *NOS1* (g.117803515C > T; rs1879417) and *NOS2* (c.1823C > T; rs2297518 and c.-227G > C; rs10459953) and the occurrence of a stroke. The SNPs were determined using the TaqMan^®^ Allelic Discrimination Assay in 107 patients with strokes and 107 age- and sex-matched individuals who had not experienced cerebrovascular accidents. The T alleles of the rs4880 were positively correlated with a stroke (bootstrap OR 1.31; 1.07–1.59 95% CI). In the case of the rs713041, an association with the T allele was found (bootstrap OR 1.36; 1.12–1.67). In addition, the occurrence of a stroke was associated with the presence of the C allele of the rs1879417 (bootstrap OR 1.32; 1.09–1.61). We also found that the C/C genotype and C allele of the rs2297518 increased the risk of a stroke (bootstrap ORs 7.00; 4.34–11.29 and 4.96; 3.88–6.34, respectively). Moreover, the C allele of the rs10459953 was associated with an increased occurrence of this disease (bootstrap OR 1.31; 1.08–1.60). These results indicated that genetics variants in the *SOD2*, *GPX4*, *NOS1* and *NOS2* might be associated with susceptibility to strokes in the Polish population.

## 1. Introduction

According to the World Health Organization, ischemic stroke is the second most common cause of death in the world, mostly affecting people over 40. Population aging and adverse lifestyle changes could increase the number of strokes and their implications in the near future [1]. Stroke leads to energy deficits of nerve tissue, which are clinically manifested focal or global neurological deficits, including: paresis, disorders of the cerebellar, body axis control and extrapyramidal, as well as cognition impairment and depressive syndromes [2]. Important social consequences caused by cerebrovascular disease cause the implementation of general strategies to reduce the risk of a stroke. To this end, modifiable risk factors of a stroke should be eliminated, and associated diseases properly treated, including smoking, high blood pressure, diabetes, physical inactivity and obesity, atrial fibrillation (AFib) or other heart disease, carotid or other artery disease, certain blood disorders, and excessive alcohol intake [3]. To reduce the degree of disability and mortality is an essential, quick, intensive, and effective implementation of treatment for patients in the early stages of a stroke and in the introduction of stroke rehabilitation. Risk of subsequent stroke should be reduced by using effective secondary prevention methods [4]. A comprehensive approach to the prevention and treatment of a stroke allows significantly reductions in the medical and social consequences of this disease. In the United States, where this strategy enables reducing the incidence of a stroke by almost 40% and mortality by 30%, it is currently only the fifth most common cause of death [5].

In the hemodynamic mechanism, impaired brain perfusion leads to ischemic stroke, which is a result of critical vasoconstriction or vessel obstruction. Cerebral circulation in patients with cerebral vasoconstriction is maintained due to cerebral autoregulation, as well as collateral blood circulation from the Willis circle and other sources [6]. In the ischemia core, the accumulation of lactate and an increase in CO_2_ pressure cause acidosis and vasodilatation–luxury perfusion. Acidosis actuates an increase in H^+^ concentration, which leads to an enhancement of superoxide anion (O^−^) dismutation to hydroxyl peroxide (H_2_O_2_) or the peroxyl radical (a more reactive form) [7].

An inflammatory process and oxidative stress are fundamental mechanisms of post-stroke brain damage [8]. Brain ischemia leads to O_2_ and glucose depletion. Reduced ATP synthesis causes inhibition of the sodium potassium pump, subsequent membrane depolarization, and an influx of Ca^2+^ ions [9]. As a result of calcium accumulation, many Ca^2+^-dependent enzymes are activated (nucleases, NO synthetases, proteases), which are the basic mechanism of cell damage. Singularly, brain susceptibility to oxidative damage is associated with a high consumption of oxygen under basal conditions, high concentrations of peroxidizable lipids, and high levels of Fe^2+^, which is a substrate in the Fenton reaction. The particular sensitivity of brain tissue to oxidative stress is due to high oxygen demand and augmented mitochondrial density [10].

Cell death and tissue damage are consequences of the accumulation of reactive oxygen species (ROS) and insufficient ability of antioxidant mechanisms. Cellular effects of ROS include protein denaturation, damage of the cytoskeletal structure, nucleic acid modification, lipid peroxidation, and enzyme inactivation [11]. ROS are characterized by vascular activity due to their effects on cerebral blood flow. Superoxide anions, peroxynitrite (ONOO^−^), and hydrogen peroxide act to increase endothelial permeability, vasodilation, and platelet aggregation. Furthermore, ROS cause changes in the reactivity of vasodilators, as well as focal lesions generation in endothelial cell membranes [12]. After ischemia/reperfusion, ROS are generated as a result of mitochondrial disfunction, activation of cyclooxygenase-2 (COX-2), nNOS and N-methyl-d-aspartate (NMDA) receptors, and the oxidation of catecholamines [13]. In turn, activation of a-amino-3-hydroxy-5-methyl-4-isoxazolepropionic acid (AMPA) receptors stimulates O-production, which, in reactions with NO, generate highly reactive ONOO^−^ [14]. 

Based on the above findings, oxidative stress can be directly involved in stroke pathogenesis and could be able to constitute a novel target for stroke therapy in the future. We hypothesize that single nucleotide polymorphisms (SNPs) in the *SOD2*, *CAT*, *GPX4*, *NOS1* and *NOS2* genes might be associated with altered susceptibility to oxidative stress and stroke development. In order to test our hypothesis, we have searched for the association between six polymorphisms: c.47T > C (rs4880) in the *SOD2* gene, c.-89A > T (rs7943316) in the *CAT* gene, c.660T > A (rs713041) in the *GPX4* gene, g.117803515C > T (rs1879417) in the *NOS1* gene, c.1823C > T (rs2297518) and c.-227G > C (rs10459953) in the *NOS2* gene and the risk of stroke. 

## 2. Materials and Methods

### 2.1. Ethics

An approval of the study was obtained from the Bioethics Committee of the Faculty of Biology and Environmental Protection of the University of Lodz, Poland (approval no. 28/2015). Written informed consent and an approval form for genetic analysis were obtained from all participants, in accordance with the Declaration of Helsinki.

### 2.2. Patients and Blood Sample Collection

A total of 214 subjects, including 107 patients with stroke and 107 age- and sex-matched individuals without stroke (controls) were recruited from the Neurorehabilitation Department of the 3rd General Hospital in Lodz, Poland, in the years 2015–2017, which has been described previously [15]. The cerebral ischemic event in each patient had been documented using computed tomography (CT) of the brain. Neurological and CT findings was interpreted by two or more independent and experienced neurologists. All the patients had been diagnosed with an ischemic stroke. In present study, we did not include patients who had had other types of strokes. Besides, among the selected individuals, the people with a history of cranial trauma, cerebral hemorrhage, atrial fibrillation, other major sources of cardioembolism, coagulation disorders, tumors, chronic inflammatory diseases, and autoimmune diseases were excluded from the study. 

All study participants were Caucasian and were recruited at the same time from the same demographic area, i.e., central Poland. The control subject had no clinical evidence of a stroke nor other cardiovascular disease. Additionally, there was no confirmation of a history of stroke for all family members (the exclusion criteria were the same as in the study group). Moreover, the patients and the control group were not related, and no one reported any genetic diseases. 

Approximately 5 mL of venous blood was drawn from each participant in the morning after 7–9 h of fasting and stored according to the same protocol. 

### 2.3. SNPs Selection and Analysis

We selected 6 potentially functional SNPs of five crucial oxidative stress-related genes, using the public domain of the single nucleotide polymorphism database (dbSNP) at the National Center for Biotechnology Information (NCBI, http://www.ncbi.nlm.nih.gov/snp; accessed on 01 July 2020) and the available literature. We selected SNPs with known genotype distribution in the European population. Our choice was mainly determined by a potential biological significance of the SNPs resulting from their location (detailed description is included in the Discussion section). The most important information about the studied polymorphisms is shown in Table 1.

Genomic DNA was isolated from peripheral blood collected in tubes containing EDTA, using a SaMag™ System (Sacace Biotecnologies Srl, Vicenza, Como, Italy) and SaMag™ Blood DNA Extraction Kit according to the instrument manufacturer’s instructions. DNA concentrations were determined by the spectrophotometric measurement of absorbance at 260 nm and the purities were calculated by A260/A280 ratio using a Bio-Tek Synergy HT Microplate Reader (Bio-Tek Instruments, Winooski, VT, USA). 

The Taq-Man^®^ SNP Genotyping Assays were performed according to the manufacturer protocol (Life Technologies, Carlsbad, CA, USA). The Taq-Man Assay IDs and thermal cycling conditions for amplifying PCR products are presented in Table 2. All reactions were carried out in a thermal cycler CFX96™ Real- Time PCR Detection System (BIO-RAD, Hercules, CA, USA). The genotypes were determined automatically based on dye-component fluorescent emission data depicted in the X–Y scatter-plot of the CFX Manager ^TM^ Software (version 3.1).

### 2.4. Statistical Analysis

Hardy–Weinberg equilibrium was checked using a *χ*^2^ test to compare the observed genotype frequencies with the expected frequencies among the case and control subjects. The *χ*^2^ analysis was also used to test the significance of the differences between distributions of genotypes and alleles in stroke patients and controls. Unconditional, multiple logistic regression analyses (codominant, dominant, and recessive models) were used to obtain the crude odds ratio (OR) and its corresponding 95% confidence interval (CI) with *p*-values for the risk of stroke. Additionally, *p*-values obtained for gene–gene analyses were corrected for multiple testing using the Bonferroni correction. The significant outcomes were further validated with the use of the bootstrap-boosted multiple logistic regression (resampling with replacement, 10,000 iterations). This was intended to overcome any possible bias related to relatively low sample sizes and to minimize the possible risk of the OR outcomes revealed by a pure chance. The goodness of fit of logistic regression models showing a significant discrimination between controls and patients was estimated with the Hosmer–Lemeshow test. Haplotypes were assessed on the basis of known genotypes of two SNPs (rs2297518, rs10459953) and the SHEsisPlus software (http://shesisplus.bio-x.cn/SHEsis.html, accessed on 17 December 2020) [16] was used. Haplotypes with frequency <0.03 were excluded from the analysis. Genetic effects of inferred haplotypes and combined genotypes were analyzed in the same way as SNPs. Linkage disequilibrium (LD) was analyzed using LDpair Tool software (https://ldlink.nci.nih.gov/?tab=ldpair, accessed on 17 December 2020). Two-sided tests of statistical significance were conducted, and a *p*-value of less than 0.05 was regarded as statistically significant. The analysis of collected data was performed in Statistica 12 (Statsoft, Tulsa, OK, USA), SigmaPlot 11.0 (Systat Software Inc., San Jose, CA, USA), Resampling Stats Add-in for Excel v.4 (Arlington, VA, USA) and StudSize3.02 (CreoStat HB, Västra Frölunda, Sweden; used for power analysis).

## 3. Results

### 3.1. Single Genotypes of SOD2, CAT, GPX4, NOS1, NOS2 Polymorphisms and Stroke Risk

For each SNP, the distribution of gene variants and alleles in stroke cases and controls, and the crude ORs and 95% CIs for stroke risk are provided in Table 3, Table 4, Table 5, Table 6 and Table 7. The OR values and the corresponding *p*-values for the genotypes/alleles increasing the risk of stroke are shown in red, and in blue are the genotypes/alleles with a protective effect.

As shown in the Table 3, Table 4, Table 5, Table 6 and Table 7, the genotype frequencies were all in agreement with the Hardy–Weinberg equilibrium calculated for the cases (*p* > 0.05; data not shown), and the differences in the frequency distributions of genotypes of the c.47T > C—*SOD2*, c.660T > C—*GPX4*, g.117803515C > T—*NOS1*, c.1823C > T and c.-227G > C—*NOS2* SNPs between the cases and controls were statistically significant (*p* < 0.05). 

*The c.47T > C (rs4880) polymorphism of the SOD2 gene and stroke risk (Table 3).*

The T allele of the c.47T > C—*SOD2* SNP were positively corelated with stroke (bootstrap crude OR 1.31; 1.07–1.59 95% CI; statistical power (SP) 0.490), while the C allele showed a negative correlation (bootstrap crude OR 0.76; 0.62–0.93 95% CI; SP 0.582). 

*The c.660T > A (rs713041) polymorphism of the GPX4 gene and stroke risk (Table 4).*

In the case of the c.660T > C—*GPX4* SNP, an association with the T allele was found (bootstrap crude OR 1.36; 1.12–1.67 95% CI; SP 0.486). On the other hand, the C allele reduced the risk of a stroke (bootstrap crude OR 0.74; 0.60–0.90 95% CI; SP 0.685). 

*The g.117803515C > T (rs1879417) polymorphism of the NOS1 gene and stroke risk (Table 5).*

In addition, the occurrence of stroke was positively correlated with the presence of the C allele of the g.117803515C > T—*NOS1* (bootstrap crude OR 1.32; 1.09–1.61 95% CI; SP 0.436), whereas the C/T genotype and T allele (bootstrap crude ORs 0.61; 0.46–0.81 95% CI; SP 0.949 and 0.76; 0.62–0.92 95% CI; SP 0.602, respectively) demonstrated a protective effect against this disease. 

*The c.1823C > T (rs2297518) polymorphism in the NOS2 gene and stroke risk (Table 6).*

We also found that the C/C genotype and C allele of the c.1832C > T—*NOS2* SNP were positively correlated with an increased risk of a stroke (bootstrap ORs 7.00; 4.34–11.29 95% CI; SP 0.472 and 4.96; 3.88–6.34; SP 0.902, respectively), while the T allele decreased this risk (bootstrap crude OR 0.20; 0.15–0.26 95% CI; SP 0.999). 

*The c.-227G > C (rs10459953) polymorphism in the NOS2 gene and stroke risk (Table 7).*

We detected that the G/G genotype and G allele of the c.-227G > C—*NOS2* SNP were associated with a decreased occurrence of a stroke (bootstrap crude ORs 0.69; 0.51–0.94 95% CI; SP 0.710 and 0.77; 0.63–0.93; SP 560, respectively). Moreover, the C allele was associated with an increased occurrence of this disease (bootstrap crude ORs 1.31; 1.08–1.60 95% CI; SP 0.414). 

*The c.-89A > T (rs7943316) polymorphism of the CAT gene and no stroke risk (Supplementary Table S1).*

Our results show that the c.-89A > T—CAT SNP was not significantly associated with a stroke. 

### 3.2. Association Between Combined Genotypes of SOD2, CAT, GPX4, NOS1, NOS2 Polymorphisms and Stroke Risk (Gene–Gene Interaction)

In order to determine whether the combined effect of two SNPs in oxidative stress-related genes modify the risk of developing a stroke, we also analyzed combinations of multilocus genotypes (Table 8). The presence of the T/C-T/C combined genotype of the rs4880-rs713041 SNPs was associated with a reduced risk of a stroke (bootstrap OR 0.64; 0.47–0.86 95% CI; SP 0.826). In addition, the presence of the G/G-T/C genotype of the rs10459953-rs4880 SNPs was associated with a decreased occurrence of a stroke (bootstrap crude OR 0.66; 0.47–0.93 95% CI; SP 0.716). Furthermore, we revealed that the C/C-T/C combined genotype of the rs2297518-rs713041 increased the risk of a stroke (bootstrap crude OR 4.30; 1.56–11.83 95% CI; SP 0.352. In addition, we observed that the C/T-T/C and C/T-C/C combined genotypes of the rs1879417-rs4880 SNPs were associated with a decreased risk of stroke (bootstrap crude ORs 0.59; 0.44–0.79; 95% CI; SP 0.925 and 0.56; 0.37–0.86; SP 0.834, respectively). Then, the presence of the C/T-T/T combined genotype of the rs1879417-rs7943316 SNPs reduced the risk of a stroke development (bootstrap crude OR 0.59; 0.40–0.86 95% CI; SP 0.848. Additionally, the presence of the C/T-T/C and C/T-C/C combined genotypes of the rs1879417-rs713041 SNPs reduced the risk of stroke development (bootstrap crude ORs 0.56; 0.40–0.77 95% CI; SP 0.942 and 0.66; 0.46–0.94; SP 0.699, respectively. Moreover, the C/T-G/G genotype of the rs1879417-rs10459953 SNPs caused a decrease in the risk of a stroke (bootstrap crude OR 0.53; 0.36–0.77 95% CI; SP 0.935). The C/T-C/C combined genotype of the rs1879417-rs2297518 SNPs was linked with an increased risk of stroke occurrence (bootstrap crude OR 2.66; 1.67–4.25 95% CI; SP 0.472). 

### 3.3. Haplotypes and Stroke Occurrence

In this study, we also investigated the association between the occurrence of stroke and haplotypes of the c.1823C > T and the c.-227G > C polymorphisms of the *NOS2* gene. The distribution of such haplotypes is shown in Table 9. The CG and CC haplotypes was associated with an increased occurrence of a stroke (crude ORs; 4.37; 2.71–7.04 95% CI and 9.98; 5.68–17.53, respectively), while TG and TC haplotypes were associated with decreased occurrence of this disease (crude ORs 0.10; 0.06–0.17 95% CI and 0.11; 0.06–0.23, respectively). LD analysis revealed that rs2297518 and rs10459953 are in linkage equilibrium (D’: 0.724, *R*^2^: 0.0796, *p* < 0.0001).

## 4. Discussion

Ischemic stroke, the most common type of a stroke, is a complex multifactorial disease caused by the interaction between environmental risk factors and genetic predisposition [17,18,19]. To date, numerous case-control studies have confirmed the association between SNPs and a stroke [20,21,22,23,24,25,26]. Ischemic strokes occurring in the carotid circulation are the most common type of an ischemic stroke, accounting for approximately 85% of all cases [27]. Growing evidence supports the essential role of oxidative stress in the development of atherosclerosis, and among them the occurrence of a stroke [28,29,30,31]. Numerous studies have shown that excessive oxidative stress, which is caused by genetic predisposition/susceptibility and environmental stress, results in the excessive production of reactive oxygen species, inflammation in the arterial atheromatous plaque, unstable atherosclerotic plaque formation, and eventually an ischemic stroke onset [29,32,33]. Furthermore, the pathophysiological role of ROS has been also intensively studied in in vitro and in vivo models of chronic neurodegenerative diseases such as Alzheimer’s disease (AD), and models of syndromes associated with rapid nerve cell loss as occurring in a stroke [34,35,36]. Functional oxidative modifications of cellular proteins, lipids, and DNA, both reversible and irreversible, are a causal step in cellular dysfunction and may modulate survival signaling cascades. Cells have a variety of defense mechanisms that intercept free radicals to prevent or limit intracellular damage and ameliorate the harmful effects of ROS, including antioxidant enzymes, such as catalase, superoxide dismutase (SOD) or glutathione peroxidase (GPx) [37,38]. Results obtained on transgenic animals in which the expression of pro-oxidant or antioxidant enzymes is genetically disrupted (knock-out mice) or enhanced (transgenic mice) showed the pathogenetic effects of a disturbed oxidative homeostasis in an ischemic brain [39,40,41]. 

The role of genetic factors involved in a stroke has extensively been studied. Several reports showed that genetic risk factors had an important role in the severity of a stroke phenotype. Genome-wide association studies (GWAS), based on a high-density single nucleotide polymorphism genotyping array, have identified several chromosomal regions/ gene loci that are associated with ischemic stroke risk. To date, a stroke can be genetically associated with multiple susceptibility loci, including 9p21 (*CDKN2A*/*CDKN2B*/*ANRIL*), 7p21 (*HDAC9*), 6p21.1, 4p25 (*PITX2*), 16q22 (*ZFHX3*), 9q34 (*ABO*) [17]. Moreover, recent studies have identified several predisposing genes that are also corelated with an ischemic stroke risk, such as *LTC4S*, *ALOX5*, *APOA1*, *APOB* [42,43]. These findings show the heterogenous and complex genetic nature of a stroke. The genotypic approach will provide better understanding and identification of the underlying genetic defects, and in the future, will enhance the possibility of medical intervention using conventional pharmacological approaches or gene therapy. 

As mentioned, oxidative stress may have a pivotal role in the development of a stroke. It is considered that the level of oxidative stress is influenced not only by conventional risk factors, i.e., hypertension and hyperlipidemia [44,45], but also inherited or acquired variations in oxidative stress-related genes. It was shown that genetic variants, such as single nucleotide polymorphisms (SNPs), which have functional effects on oxidative stress pathways, may represent unmodifiable, lifelong risk factors, and gene–environment interactions may further contribute to a cerebrovascular disease risk [46,47]. Functionality of a protein is determined by several factors, including variation in its gene, because it determines many sequence-specific interactions important for the expression of the gene [48]. The present study was undertaken to detect the potential association of six SNPs in oxidative stress-related genes: *SOD2* (c.47T > C; rs4880), *CAT* (c.-89A > T; rs7943316), *GPX4* (c.660T > A; rs713041), *NOS1* (g.117803515C > T; rs1879417) and *NOS2* (c.1823C > T; rs2297518 and c.-227G > C; rs10459953) and the occurrence of a stroke. 

In the present work, we found a significant association between rs4880-SOD2 and the occurrence of a stroke. *SOD2* gene (Gene ID: 6648), encodes mitochondrial manganese superoxide dismutase (MnSOD) and is located in the region 6q25. It has been demonstrated that the SOD2 is the only antioxidant enzyme known to be present within the mitochondria, which has important implications because it is a major site for the production of ROS during normal cellular metabolism [49]. The human *SOD2* gene is highly polymorphic; at least 26,687 of its SNPs have been registered in the public domain of the NCBI dbSNP. Among them, rs4880 is one of the most important and it plays an essential role in various kinds of diseases. It has been demonstrated that this SNP is associated with an increased risk of breast, prostate, bladder and lung cancers, carotid atherosclerosis, and cardiomyopathy [50,51,52,53,54]. The rs4880 SNP is located in exon 2 and substitutes a C > T at position 47 in the coding sequence, which causes change of alanine to valine at the amino acid position 16 (p.Val16Ala) and may potentially result in functional alteration [55]. Research has shown that the Val allele of this SNP results in a reduced expression and production of an unstable mRNA, which affects the import of SOD2 into the mitochondrion [56]. To the best of our knowledge, no previous studies have investigated the role of this SNP in stroke development. We have observed, for the first time, that the occurrence of a stroke is positively corelated with the T allele. 

Furthermore, we did not find any association between second potentially functional polymorphism c.-89A > T (rs7943316) in the *CAT* gene and the occurrence of a stroke. Our findings are in contrast with the studies conducted on the Russian population [57], where the authors observed that the A/A genotype is associated with an increased risk of a cerebral stroke (CS) in hypertensive males (OR = 1.77 95% CI 1.01–3.07). Our results also show that the c.660T > A (rs713041) of the *GPX4* gene may be associated with stroke occurrence. The protein encoded by *GPX4* gene (Gene ID: 2879) belongs to the glutathione peroxidase family, members of which catalyze the reduction of hydrogen peroxide, organic hydroperoxides and lipid hydroperoxides, and thus protect cells against oxidative damage. *GPX4* gene is located on chromosome 19 (19p13.3) and, as provided by NCBI dbSNP, 2232 SNPs in this gene have been registered. The rs713041 is a silent mutation, located in exon 7 that substitutes T for A at position 660, which changes the amino acid at position 220 from leucine to leucine (p.Leu220=). Foster et al. have observed that this SNP is involved in the modulation of the GPX4 synthesis by altering the affinity of the selenocysteine insertion machinery for its SECIS element [58]. Other studies have shown that the C variant of the *GPX4* gene was stronger than the T variant at driving biosynthesis of a selenoprotein reporter [59]. In this matter, our findings are similar to the studies conducted on a Russian population [60]—we observed an association between a stroke and the T allele. It was also shown that this SNP was associated with mortality in patients with breast cancer [61] and colorectal cancer susceptibility [59]. 

As commonly known, brain ischemia initiates a complex cascade of metabolic events, several of which involve the generation of nitrogen and oxygen free radicals. It has been shown that nitric oxide was implicated in the neurotoxicity associated with a stroke and other neurodegenerative diseases, because the alterations in the NO signaling play a key role in diverse neurodegenerative-associated processes such as neuronal cell death, necrosis, apoptosis, and autophagy [62,63,64]. Moreover, numerous studies have shown that ischemia is associated with a large increase in nitric oxide synthase 1 (NOS1; nNOS) activity in neurons and possibly in glia, next increased NOS3 (eNOS) activity in the vascular endothelium, and later caused an increase in NOS2 (iNOS) activity in a range of cells, including infiltrating neutrophils and macrophages, activated microglia and astrocytes [64,65,66]. Additionally, it is interesting that NO has a dual function/role, including neuroprotection and neurotoxicity during ischemia reperfusion. In general, nNOS and iNOS play a neuronal injury role in the early and late stage of an ischemic stroke, while the activation of eNOS mainly exerts neuroprotection effects [67]. The toxic effects of NO produced by iNOS and nNOS are mainly due to the production of nitrates and the release of free radicals, which directly damages mitochondrial enzymes, nucleic acids and proteins, leading to neuronal cell death [34,67,68,69]. On the other hand, neuroprotective effects of NO produced by eNOS are achieved primarily by regulating vasodilatation and angiogenesis [67,70,71]. The NO donors as well as NOS inhibitors also have a neuroprotective effect, which is confirmed by studies conducted on experimental models of ischemic stroke [66,72,73]. To summarize, the beneficial or harmful role of NO in the brain tissue of an ischemic stroke is mainly dependent on the cell type, NO concentration, and/or microenvironment of ischemia [67,74,75]. Better understanding of the mechanism of imbalanced NO metabolism contributing to the neuronal cell death may open new perspectives in the prevention and treatment of neurodegenerative diseases, among them, ischemic stroke.

It has been indicated that the activity of nitric oxide synthases can be modified by polymorphisms of their genes [76,77]. In the *NOS1* gene (Gene ID: 4842; also known as *nNOS*) located on the long arm of chromosome 12, at position 12q24.22, 38,964 SNPs have been described. We chose for genotyping rs1879417 (g.117803515C > T) SNPs located in intron. Polymorphisms in intron sequences can affect the mRNA/protein splicing process, resulting in the formation of different isoforms of a protein [78,79,80]. These SNPs has not been studied in a stroke so far. Our case-control studies have shown for the first time that the subjects carrying C alleles had a higher risk of developing a stroke than subjects without that allele. In contrast, in our previous studies, we did not find any correlation between the genotypes/alleles of this polymorphism and the occurrence of depressive disorders (DD) [81]. In other studies, *NOS1* rs1879417 was also found to be associated with a lower cognitive performance [82]. In the present study, we have additionally investigated, the prevalence of two SNPs in *NOS2* gene (c.1823C > T; rs2297518) and c.-227G > C; rs10459953), and the association between these genetic polymorphisms and stroke risk. *NOS2* gene (Gene ID: 4843, also known as iNOS) is present on the human chromosome 17q11.2–12. The rs2297518 is located in exon 16 and causes an amino acid substitution from serine to leucine at the amino acid position 608 (p.Ser608Leu). It was shown that this SNP is associated with increased NOS2 activity (alters protein function) and confers higher NO production based on the A allele [83]. This polymorphism has been found to be associated with inflammatory bowel disease, gastric cancer and additionally, with vulnerable plaque among subjects with a high stroke risk [83,84,85]. Our study also showed an association of the C/C genotype and C allele with stroke occurrence. The second SNP rs10459953 is located in the 5′-untranslated region and, because of the location, it might affect the translation rate of the mRNA and hence affect the iNOS protein level [86,87]. It was previously shown to be associated with prostatic volume in benign prostatic hyperplasia and type 2 diabetes mellitus and diabetic nephropathy [88,89]. Moreover, in our previous research, we confirmed that the G/C and G/G genotypes of the rs10459953 were associated with the development of depression in Polish women [81]. In the presented work, in turn, we observed that this SNP may be associated with stroke occurrence, because the C allele was positively correlated with an increased risk of this disease. Furthermore, our data indicate for the first time that stroke susceptibility may be modulated not only by single locus with genetic main effects, but also by epistatic (gene–gene) interactions in oxidative stress-related genes studied in this paper. Our results show a strong association between a number of two-gene combinations and an increased risk of a stroke: rs4880-rs713041, rs10459953-rs4880, rs2297518-rs713041, rs1879417-rs4880, rs1879417-rs7943316, rs1879417-rs713041, rs1879417-rs10459953, rs1879417-rs2297518. 

We are aware that the presented research has some limitations because the sample size was relatively small. However, to minimalize the risk of obtaining false positive results, two resampling approaches were employed. Furthermore, we want to clearly emphasize that our case-control study is preliminary, limited to a single population, which in turn gives the possibility that the results may not be duplicated in other populations (the statistical power for some genotypes/alleles was below 80%). Additionally, it would also be interesting to determine the levels of enzymes encoded by the studied genes in the serum. However, the above limitations follow from the specificity of the target material and its limited accessibility. To summarize, further studies are needed on a larger group of patients and other populations before the relationship between the studied SNPs and the risk of a stroke can be finally established. Therefore, our results should be interpreted with caution and considered as preliminary—they may guide the direction of research for other teams. 

## 5. Conclusions

In conclusion, our results showed that the genetics variants in the *SOD2* (c.47T > C; rs4880), *GPX4* (c.660T > A; rs713041), *NOS1* (g.117803515C > T; rs1879417) and *NOS2* (c.1823C > T; rs2297518 and c.-227G > C; rs10459953) genes may be associated with individual susceptibility to a stroke. This knowledge might help to identify specific molecular markers of a stroke and develop new therapeutic strategies. We postulate that the pharmacological modification of oxidative lesions would be one of the most promising direction for the development of future stroke therapy.

## Figures and Tables

**Table 1:** Basic information of the *SOD2*, *CAT*, *GPX4*, *NOS1* and *NOS2* polymorphisms.

Gene	Region	NCBI db SNP ID (rs Number)	Position in g.DNA or c.DNA	Base Change	Amino Acid Change	Methods of Genotyping	MAF *
***SOD2***	exon	rs4880	c.47	T > C	p.Val16Ala	TaqMan^®^ SNP Genotyping Assays	C: 0.466
***CAT***	UTR-5	rs7943316	c.-89	A > T	-		A: 0.331
***GPX4***	exon	rs713041	c.660	T > C	p.Leu220=		T: 0.449
***NOS1***	intron	rs1879417	g.117803515	C > T	-		C: 0.449
***NOS2***	exon	rs2297518	c.1823	C > T	p.Ser608Leu		T: 0.232
	UTR-5	rs10459953	c.-227	G > C	-		C: 0.360

*—minor allele frequency (MAF) in European population.

**Table 2:** TaqMan^®^ SNP Genotyping Assays used in this study.

Polymorphism	Assay ID	Location	PCR Conditions			
				Time	Temperature	
rs4880	C___8709053_10	Chr.6: 159692840	AmpliTaq Gold Enzyme Activation	10 min	95 °C	
rs7943316	C___1883210_10	Chr.11: 34438925				
rs713041	C___2561693_20	Chr.19: 1106616	Denature	15 s	92 °C	40 cycles
rs1879417	C__11754652_10	Chr.12: 117365710				
rs2297518	C__11889257_10	Chr.17: 27769571	Anneal/ Extend	60 s	60 °C	
rs10459953	C___2593687_10	Chr.17: 27800492				

**Table 3:** Distribution of genotypes and alleles of the c.47T > C (rs4880) polymorphism in the *SOD2* gene and odds ratios (ORs) with 95% confidence intervals (95% CIs) in patients with stroke and controls.

Genotype/Allele	Control (*n* = 107)		Stroke (*n* = 107)		Crude OR (95% CI)	*p*
	Number	Frequency	Number	Frequency		
T/T	2	0.019	23	0.215	14.38 (3.30–62.72) * ^B^ 10.00 (0.07–1377.03)^0.144^ ^Cv^ 14.38 (3.30–62.72)	<0.001 0.359 <0.001
T/C	71	0.664	57	0.533	0.58 (0.33–1.00)	0.052
C/C	34	0.318	27	0.252	0.73 (0.40–1.32)	0.290
*χ*^2^ = 19.975; ***p* < 0.0001**						
T	75	0.350	103	0.481	1.72 (1.17–2.54) * 1.31 (1.07–1.59)^0.490^ 1.72 (1.17–2.54)	0.006 0.009 0.006
C	139	0.650	111	0.519	0.58 (0.39–0.86) * 0.76 (0.62–0.93)^0.582^ 0.58 (0.39–0.86)	0.006 0.006 0.006

* crude OR means OR calculated with conventional logistic regression; for the significant outcomes, the superscript ^B^ means the bootstrap-boosted OR (resampling with replacement, 1000 iterations); ^Cv^ means the cross-validated OR. Statistical power (1-β) for significant comparisons is given in superscripts. *p* < 0.05 along with the corresponding ORs are in red (for the genotypes/alleles increasing the risk of stroke) or in blue (for the genotypes/alleles with a protective effect).

**Table 4:** Distribution of genotypes and alleles of the c.660T > A (rs713041) polymorphism in the *GPX4* gene and odds ratios (ORs) with 95% confidence intervals (95% CIs) in patients with stroke and controls.

Genotype/Allele	Control (*n* = 107)		Stroke (*n* = 107)		Crude OR (95% CI)	*p*
	Number	Frequency	Number	Frequency		
T/T	2	0.019	22	0.206	13.59 (3.11–59.43) * ^B^ 9.43 (0.07–1247.38)^0.141^ ^Cv^ 13.59 (3.11–59.43)	0.001 0.368 0.001
T/C	64	0.598	55	0.514	0.71 (0.41–1.22)	0.216
C/C	41	0.383	30	0.280	0.63 (0.35–1.11)	0.111
*χ*^2^ = 19.052; ***p* < 0.0001**						
T	68	0.318	99	0.463	1.85 (1.25–2.74) * ^B^ 1.36 (1.12–1.67)^0.486^ ^Cv^ 1.85 (1.25–2.74)	0.002 0.003 0.002
C	146	0.682	115	0.537	0.54 (0.37–0.80) * ^B^ 0.74 (0.60–0.90)^0.685^ ^Cv^ 0.54 (0.37–0.80)	0.002 0.003 0.002

* crude OR means OR calculated with conventional logistic regression; for the significant outcomes, the superscript ^B^ means the bootstrap-boosted OR (resampling with replacement, 1000 iterations); ^Cv^ means the cross-validated OR. Statistical power (1-β) for significant comparisons is given in superscripts. *p* < 0.05 along with the corresponding ORs are in red (for the genotypes/alleles increasing the risk of stroke) or in blue (for the genotypes/alleles with a protective effect).

**Table 5:** Distribution of genotypes and alleles of the g.117803515C > T (rs1879417) polymorphism in the *NOS1* gene and odds ratios (ORs) with 95% confidence intervals (95% CIs) in patients with stroke and controls.

Genotype/Allele	Control (*n* = 107)		Stroke (*n* = 107)		Crude OR (95% CI)	*p*
	Number	Frequency	Number	Frequency		
C/C	1	0.009	28	0.262	37.57 (5.01–282.03) * ^B^ 76.21 (0.08–71181.91)^0.070^ ^Cv^ 37.57 (5.01–282.04)	<0.001 0.214 <0.001
C/T	74	0.692	49	0.458	0.38 (0.22–0.66) * ^B^ 0.61 (0.46–0.81)^0.949^ ^Cv^ 0.38 (0.22–0.66)	0.001 0.001 0.001
T/T	32	0.299	30	0.280	0.91 (0.51–1.65) *	0.763
*χ*^2^ = 30.284; ***p* < 0.0001**						
C	76	0.355	105	0.491	1.75 (1.19–2.58) * ^B^ 1.32 (1.09–1.61)^0.436^ ^Cv^ 1.75 (1.19–2.58)	0.005 0.005 0.005
T	138	0.645	109	0.509	0.57 (0.39–0.84) * ^B^ 0.76 (0.62–0.92)^0.602^ ^Cv^ 0.57 (0.39–0.84)	0.005 0.005 0.005

* crude OR means OR calculated with conventional logistic regression; for the significant outcomes, the superscript ^B^ means the bootstrap-boosted OR (resampling with replacement, 1000 iterations); ^Cv^ means the cross-validated OR. Statistical power (1-β) for significant comparisons is given in superscripts. *p* < 0.05 along with the corresponding ORs are in red (for the genotypes/alleles increasing the risk of stroke) or in blue (for the genotypes/alleles with a protective effect).

**Table 6:** Distribution of genotypes and alleles of the c.1823C > T (rs2297518) polymorphism in the *NOS2* gene and odds ratios (ORs) with 95% confidence intervals (95% CIs) in patients with stroke and controls.

Genotype/Allele	Control (*n* = 107)		Stroke (*n* = 107)		Crude OR (95% CI)	*p*
	Number	Frequency	Number	Frequency		
C/C	7	0.065	81	0.757	44.51 (18.38–107.78) * ^B^ 7.00 (4.34–11.29)^0.472^ ^Cv^ 44.51 (18.38–107.79)	<0.001 <0.001 <0.001
C/T	32	0.299	24	0.224	0.68 (0.37–1.25) *	0.215
T/T	68	0.636	2	0.019	0.01 (0.003–0.05) * ^B^0.04 (0.001–7.17)^0.999^ ^Cv^0.01 (0.003–0.05)	<0.001 0.219 <0.001
*χ*^2^ = 125.599; ***p* < 0.0001**						
C	46	0.215	186	0.869	24.26 (14.51–40.57) * ^B^ 4.96 (3.88–6.34)^0.902^ ^Cv^ 24.26 (14.51–40.57)	<0.001 <0.001 <0.001
T	168	0.785	28	0.131	0.04 (0.03–0.07) * ^B^ 0.20 (0.15–0.26)^0.999^ ^Cv^ 0.04 (0.03–0.07)	<0.001 <0.001 <0.001

* crude OR means OR calculated with conventional logistic regression; for the significant outcomes, the superscript ^B^ means the bootstrap-boosted OR (resampling with replacement, 1000 iterations); ^Cv^ means the cross-validated OR. Statistical power (1-β) for significant comparisons is given in superscripts. *p* < 0.05 along with the corresponding ORs are in red (for the genotypes/alleles increasing the risk of stroke) or in blue (for the genotypes/alleles with a protective effect).

**Table 7:** Distribution of genotypes and alleles of the c.-227G > C (rs10459953) polymorphism in the *NOS2* gene and odds ratios (ORs) with 95% confidence intervals (95% CIs) in patients with stroke and controls.

Genotype/Allele	Control (*n* = 107)		Stroke (*n* = 107)		Crude OR (95% CI)	*p*
	Number	Frequency	Number	Frequency		
G/G	44	0.411	27	0.271	0.48 (0.27–0.87) * ^B^ 0.69 (0.51–0.94)^0.710^ ^Cv^ 0.48 (0.27–0.87)	0.014 0.017 0.014
G/C	45	0.421	51	0.477	1.26 (0.73–2.15) *	0.410
C/C	18	0.168	29	0.252	1.84 (0.95–3.56) *	0.071
*χ*^2^ = 7.020; ***p* = 0.0299**						
G	133	0.621	105	0.491	0.59 (0.40–0.86) * ^B^ 0.77 (0.63–0.93)^0.560^ ^Cv^ 0.59 (0.40–0.86)	0.007 0.008 0.007
C	81	0.379	109	0.509	1.71 (1.16–2.51) * 1.31 (1.08–1.60)^0.414^ 1.71 (1.16–2.51)	0.007 0.006 0.007

* crude OR means OR calculated with conventional logistic regression; for the significant outcomes, the superscript ^B^ means the bootstrap-boosted OR (resampling with replacement, 1000 iterations); ^Cv^ means the cross-validated OR. Statistical power (1-β) for significant comparisons is given in superscripts. *p* < 0.05 along with the corresponding ORs are in red (for the genotypes/alleles increasing the risk of stroke) or in blue (for the genotypes/alleles with a protective effect).

**Table 8:** Distribution of combined genotypes of the c.47T > C (rs4880) in the *SOD2* gene, c.-89A > T (rs7943316) in the *CAT* gene, c.660T > A (rs713041) in the *GPX4* gene, g.117803515C > T (rs1879417) in the *NOS1* gene, c.1823C > T (rs2297518) and c.-227G > C (rs10459953) in the *NOS2* gene polymorphisms and odds ratios (ORs) with 95% confidence intervals (95% CIs) in patients with stroke and controls.

**Genotype**	**Control (*n* = 107)**		**Stroke (*n* = 107)**		**Crude OR (95% CI)**	***p***
	**Number**	**Frequency**	**Number**	**Frequency**		
**c.-89A > T (rs7943316)—*CAT* vs. c.47T > C (p.Val16Ala)—*SOD2* (rs4880)**						
A/A-T/T	0	0	0	0	-	-
A/A-T/C	15	0.140	6	0.056	0.36 (0.14–0.98) *	0.088 ^#^
A/A-C/C	5	0.047	9	0.084	1.87 (0.61–5.79) *	0.474 ^#^
A/T-T/T	1	0.009	14	0.131	15.96 (2.06–123.68) * ^B^ 50.46 (0.05–50,144.36)^0.070^ ^Cv^ 15.96 (2.06–123.68)	0.016 ^#^ 0.265 0.016 ^#^
A/T-T/C	32	0.299	36	0.336	1.189 (0.67–2.12) *	0.804 ^#^
A/T-C/C	13	0.121	9	0.084	0.66 (0.27–1.63) *	0.603 ^#^
T/T-T/T	1	0.009	9	0.084	9.74(1.21–78.25) *	0.063 ^#^
T/T-T/C	24	0.224	15	0.140	0.56 (0.28–1.15) *	0.215 ^#^
T/T-C/C	16	0.150	9	0.084	0.52 (0.22–1.24) *	0.262 ^#^
**Genotype**	**Control (*n* = 107)**		**Stroke (*n* = 107)**		**Crude OR (95% CI)**	***p***
	**Number**	**Frequency**	**Number**	**Frequency**		
**c.-89A > T (rs7943316)—*CAT* vs. c.660T > C—*GPX4* (rs713041)**						
A/A-T/T	1	0.009	4	0.037	4.12 (0.45–37.45) *	0.374 ^#^
A/A-T/C	12	0.112	7	0.064	0.55 (0.21–1.47) *	0.415 ^#^
A/A-C/C	7	0.065	4	0.037	0.56 (0.16–1.95) *	0.589 ^#^
A/T-T/T	1	0.009	15	0.140	17.28 (2.24–133.37) * ^B^ 57.95 (0.05–62,416.10)^0.070^ ^Cv^ 17.28 (2.24–133.37)	0.012 ^#^ 0.254 0.012 ^#^
A/T-T/C	29	0.271	26	0.243	0.86 (0.47–1.60) *	0.870 ^#^
A/T-C/C	16	0149	18	0.168	1.15 (0.55–2.40) *	0.915 ^#^
T/T-T/T	0	0	3	0.028	-	-
T/T-C/T	23	0.215	22	0.206	0.95 (0.49–1.83) *	0.982 ^#^
T/T-C/C	18	0.168	8	0.075	0.40 (0.17–0.96) *	0.080 ^#^
**Genotype**	**Control (*n* = 107)**		**Stroke (*n* = 107)**		**Crude OR (95% CI)**	***p***
	**Number**	**Frequency**	**Number**	**Frequency**		
**c.47T > C (p.Val16Ala)—*SOD2* (rs4880) vs. c.660T > C—*GPX4* (rs713041)**						
T/T-T/T	0	0	3	0.028	-	-
T/T-T/C	1	0.009	16	0.150	18.64 (2.42–143.28) * 50.98 (0.05–50,200.29)0.070 18.64 (2.42–143.28)	0.010 ^#^ 0.264 0.010 ^#^
T/T-C/C	1	0.009	4	0.037	4.12 (0.45–37.45) *	0.374 ^#^
T/C-T/T	1	0.009	14	0.131	15.96 (2.06–123.68) * ^B^ 42.94 (0.05–39,012.16)^0.070^ ^Cv^ 15.96 (2.06–123.68)	0.016 ^#^ 0.279 0.016 ^#^
T/C-T/C	40	0.374	21	0.196	0.41 (0.22–0.76) * ^B^ 0.64 (0.47–0.86)^0.826^ ^Cv^ 0.41 (0.22–0.76)	0.010 ^#^ 0.004 0.010 ^#^
T/C-C/C	30	0.280	22	0.206	0.66 (0.35–1.25) *	0.366 ^#^
C/C-T/T	1	0.009	5	0.047	5.20 (0.60–45.24) *	0.254 ^#^
C/C-T/C	23	0.215	18	0.168	0.74 (0.37–1.47) *	0.623 ^#^
C/C-C/C	10	0.093	4	0.037	0.38 (0.11–1.24) *	0.204 ^#^
**Genotype**	**Control (n = 107)**		**Stroke (n = 107)**		**Crude OR (95% CI)**	***p***
	**Number**	**Frequency**	**Number**	**Frequency**		
**c.1823C > T (p.Ser608Leu)—*NOS2* (rs2297518) vs. c.47T > C (p.Val16Ala)—*SOD2* (rs4880)**						
C/C-T/T	0	0	18	0.168	-	-
C/C-T/C	4	0.037	44	0.411	17.98 (6.17–52.46) * ^B^ 5.18 (0.59–45.77)^0.278^ ^Cv^ 17.98 (6.17–52.46)	<0.001 ^#^ 0.139 ^#^ <0.001 ^#^
C/C-C/C	3	0.028	19	0.178	7.49 (2.14–26.13) * ^B^ 4.86 (0.11–216.99)^0.210^ ^Cv^ 7.49 (2.14–26.13)	0.004 ^#^ 0.415 0.004#
C/T-T/T	1	0.009	5	0.047	5.05 (0.58–43.98) *	0.266 ^#^
C/T-T/C	23	0.215	12	0.112	0.47 (0.22–1.01) *	0.103 ^#^
C/T-C/C	8	0.075	7	0.065	0.84 (0.29–2.41)	0.935 ^#^
T/T-T/T	1	0.009	0	0	-	-
T/T-T/C	44	0.411	1	0.009	0.014 (0.002–0.01) * ^B^ 0.010 (0.0001–10.45)^0.998^ ^Cv^ 0.014 (0.002–0.01)	<0.001 ^#^ 0.193 <0.001#
T/T-C/C	23	0.215	1	0.009	0.03 (0.0001–0.26) * ^B^ 0.016 (0.0001–14.32)^0.989^ ^Cv^0.03 (0.0001–0.26)	0.002 ^#^ 0.232 0.002 ^#^
**Genotype**	**Control (*n* = 107)**		**Stroke (*n* = 107)**		**Crude OR (95% CI)**	***p***
	**number**	**frequency**	**number**	**frequency**		
**c.1823C > T (p.Ser608Leu)—*NOS2* (rs2297518) vs. c.660T > C—*CAT* (rs7943316)**						
C/C-A/A	2	0.019	12	0.112	6.63 (1.45–30.40) * ^B^ 7.20 (0.04–1166.78)^0.141^ ^Cv^ 6.63 (1.45–30.40)	0.030 ^#^ 0.447 0.030 ^#^
C/C-A/T	3	0.028	44	0.411	24.21 (7.22–81.25) * ^B^ 7.17 (0.31–167.13)^0.210^ ^Cv^24.21 (7.22–81.25)	<0.001 ^#^ 0.220 <0.001 ^#^
C/C-T/T	2	0.019	25	0.234	16.01 (3.68–69.54) * ^B^ 9.97 (0.08–12,8618)^0.141^ ^Cv^ 16.01 (3.68–69.54)	<0.001 ^#^ 0.354 <0.001 ^#^
C/T-A/A	7	0.065	3	0.028	0.41 (0.10–1.64) *	0.373 ^#^
C/T-A/T	11	0.103	15	0.140	1.42 (0.62–3.26) *	0.645 ^#^
C/T-T/T	14	0.131	6	0.056	0.40 (0.15–1.07) *	0.131 ^#^
T/T-A/A	11	0.103	0	0	-	-
T/T-A/T	32	0.299	0	0	-	-
T/T-T/T	25	0.234	2	0.187	0.06 (0.01–0.27) * ^B^ 0.08 (0.0001–15.03)^0.993^ ^Cv^ 0.06 (0.01–0.27)	<0.001 ^#^ 0.348 <0.001 ^#^
**Genotype**	**Control (*n* = 107)**		**Stroke (*n* = 107)**		**Crude OR (95% CI)**	***p***
	**Number**	**Frequency**	**Number**	**Frequency**		
**c.-227G > C—*NOS2* (rs10459953) vs. c.660T > C—*GPX4* (rs713041)**						
C/C-T/T	0	0	9	0.084	-	-
C/C-T/C	14	0.131	11	0.103	0.76 (0.33–1.76) *	0.773 ^#^
C/C-C/C	4	0.037	9	0.084	2.37 (0.71–7.93) *	0.299 ^#^
G/C-T/T	2	0.019	9	0.084	4.82 (1.02–22.87) *	0.094 ^#^
G/C-T/C	23	0.215	26	0.243	1.17 (0.62–2.20) *	0.860 ^#^
G/C-C/C	20	0.187	16	0.150	0.77 (0.37–1.57) *	0.715 ^#^
G/G-T/T	0	0	4	0.037	-	-
G/G-T/C	27	0.252	18	0.168	0.60 (0.31–1.17) *	0.248 ^#^
G/G-C/C	17	0.159	5	0.047	0.26 (0.09–0.73) * ^B^ 0.45 (0.09–2.28)^0.851^ ^Cv^0.26 (0.09–0.73)	0.022 ^#^ 0.337 0.022
**Genotype**	**Control (*n* = 107)**		**Stroke (*n* = 107)**		**Crude OR (95% CI)**	***p***
	**Number**	**Frequency**	**Number**	**Frequency**		
**c.-227G > C—*NOS2* (rs10459953) vs. c.47T > C (p.Val16Ala)—*SOD2* (rs4880)**						
G/G-T/T	1	0.009	3	0.028	3.06 (0.31–29.87) *	0.560 ^#^
G/G-T/C	32	0.299	17	0.159	0.44 (0.23–0.86) * ^B^ 0.66 (0.47–0.93)^0.716^ ^Cv^ 0.44 (0.23–0.86)	0.032 ^#^ 0.018 0.032 ^#^
G/G-C/C	11	0.103	7	0.065	0.61 (0.23–1.64) *	0.548 ^#^
G/C-T/T	1	0.009	14	0.131	15.96 (2.06–123.68) * ^B^ 41.23 (0.05–36,529.68)^0.070^ ^Cv^0.44 (0.23–0.86)	0.016 ^#^ 0.283 0.016 ^#^
G/C-T/C	29	0.271	27	0.252	0.91 (0.49–1.67) *	0.940 ^#^
G/C-C/C	15	0.140	10	0.093	0.62 (0.27–1.48) *	0.496 ^#^
C/C-T/T	0	0	6	0.056	-	-
C/C-T/C	10	0.093	13	0.121	1.34 (0.56–3.21) *	0.759 ^#^
C/C-C/C	8	0.075	10	0.093	1.28 (0.48–3.37) *	0.858 ^#^
**Genotype**	**Control (*n* = 107)**		**Stroke (*n* = 107)**		**Crude OR (95% CI)**	***p***
	**Number**	**Frequency**	**Number**	**Frequency**		
**c.1823C > T (p.Ser608Leu)—*NOS2* (rs2297518) vs. c.660T > C—*GPX4* (rs713041)**						
C/C-T/T	0	0	16	0.150	-	-
C/C-T/C	5	0.047	46	0.430	15.38 (5.80–40.82) * ^B^ 4.30 (1.56–11.83)^0.352^ ^Cv^ 15.38 (5.80–40.82)	<0.001 ^#^ 0.005 <0.001 ^#^
C/C-C/C	2	0.019	19	0.178	11.34 (2.57–50.01) * ^B^ 8.12 (0.07–951.43)^0.141^ ^Cv^11.34 (2.57–50.01)	0.002 ^#^ 0.389 0.002 ^#^
C/T-T/T	1	0.009	6	0.056	6.30 (0.75–53.23) *	0.174 ^#^
C/T-T/C	17	0.159	8	0.075	0.43 (0.18–1.04) *	0.118
C/T-C/C	14	0.131	10	0.093	0.69 (0.29–1.62) *	0.625
T/T-T/T	1	0.009	0	0	-	-
T/T-T/C	42	0.393	1	0.009	0.02 (0.002–0.11) * ^B^ 0.01 (0.0001–10.98)^0.998^ ^Cv^0.02 (0.002–0.11)	<0.001 ^#^ 0.198 <0.001 ^#^
T/T-C/C	25	0.234	1	0.009	0.03 (0.004–0.23) * ^B^ 0.02 (0.0001–13.95)^0.992^ ^Cv^0.03 (0.004–0.23)	0.002 ^#^ 0.229 0.002
**Genotype**	**Control (*n* = 107)**		**Stroke (*n* = 107)**		**Crude OR (95% CI)**	***p***
	**Number**	**Frequency**	**Number**	**Frequency**		
**g.117803515C > T—*NOS1* (rs1879417) vs. c.47T > C (p.Val16Ala)—*SOD2* (rs4880)**						
C/C-T/T	0	0	1	0.009	-	-
C/C-T/C	1	0.009	18	0.168	21.44 (2.81–163.77) * ^B^ 50.55 (0.05–48,833.74)^0.070^ ^Cv^21.44 (2.81–163.77)	0.006 ^#^ 0.263 0.006 ^#^
C/C-C/C	0	0	9	0.084	-	-
C/T-T/T	1	0.009	15	0.140	17.28 (2.24–133.37) * ^B^ 49.37 (0.05–47,718.94)^0.070^ ^Cv^17.28 (2.24–133.37)	0.012 ^#^ 0.266 0.012 ^#^
C/T-T/C	48	0.449	24	0.224	0.36 (0.20–0.64) * ^B^ 0.59 (0.44–0.79)^0.925^ ^Cv^ 0.36 (0.20–0.64)	0.001 ^#^ <0.01 0.001 ^#^
C/T-C/C	25	0.234	10	0.093	0.34 (0.15–0.75) * ^B^ 0.56 (0.37–0.86)^0.834^ ^Cv^ 0.34 (0.15–0.75)	0.014 ^#^ 0.007 0.014 ^#^
T/T-T/T	1	0.009	7	0.065	7.42 (0.90–61.39) *	0.122 ^#^
T/T-T/C	22	0.206	15	0.140	0.63 (0.31–1.29) *	0.373 ^#^
T/T-C/C	9	0.084	8	0.075	0.88 (0.33–2.37) *	0.960 ^#^
**Genotype**	**Control (*n* = 107)**		**Stroke (*n* = 107)**		**Crude OR (95% CI)**	***p***
	**Number**	**Frequency**	**Number**	**Frequency**		
**g.117803515C > T—*NOS1* (rs1879417) vs. c.-89A > T—*CAT* (rs7943316)**						
C/C-A/A	0	0	6	0.056	-	-
C/C-A/T	0	0	12	0.112	-	-
C/C-T/T	1	0.009	10	0.093	10.93 (1.37–86.95) * ^B^ 37.12 (0.04–34,331.70)^0.070^ ^Cv^10.93 (1.37–86.95)	0.047 ^#^ 0.300 0.047 ^#^
C/T-A/A	11	0.103	5	0.047	0.43 (0.14–1.28) *	0.240 ^#^
C/T-A/T	33	0.308	31	0.290	0.92 (0.51–1.64) *	0.945 ^#^
C/T-T/T	30	0.280	13	0.121	0.36 (0.17–0.73) * ^B^ 0.59 (0.40–0.86)^0.848^ ^Cv^ 0.36 (0.17–0.73)	0.010 ^#^ 0.007 0.010 ^#^
T/T-A/A	9	0.084	4	0.037	0.42 (0.13–1.42) *	0.299 ^#^
T/T-A/T	13	0.121	16	0.150	1.27 (0.58–2.79) *	0.798 ^#^
T/T-T/T	10	0.093	10	0.093	1.00 (0.40–2.51) *	1.000 ^#^
**Genotype**	**Control (*n* = 107)**		**Stroke (*n* = 107)**		**Crude OR (95% CI)**	***p***
	**Number**	**Frequency**	**Number**	**Frequency**		
**g.117803515C > T—*NOS1* (rs1879417) vs. c.660T > C—*GPX4* (rs713041)**						
C/C-T/T	0	0	2	0.019	-	-
C/C-T/C	0	0	19	0.178	-	-
C/C-C/C	1	0.009	7	0.065	7.42 (0.90–61.39) *	0.122 ^#^
C/T-T/T	2	0.019	15	0.140	8.56 (1.91–38.43) * ^B^ 6.90 (0.06–768.93)^0.141^ ^Cv^8.56 (1.91–38.43)	0.010 ^#^ 0.422 ^#^ 0.010 ^#^
C/T-T/C	43	0.402	19	0.178	0.32 (0.17–0.60) * ^B^ 0.56 (0.40–0.77)^0.942^ ^Cv^ 0.32 (0.17–0.60)	0.001 ^#^ <0.01 0.001 ^#^
C/T-C/C	29	0.271	15	0.140	0.44 (0.22–0.88) * ^B^ 0.66 (0.46–0.94)^0.699^ ^Cv^ 0.44 (0.22–0.88)	0.040 ^#^ 0.023 0.040 ^#^
T/T-T/T	0	0	5	0.047	-	-
T/T-T/C	21	0.196	17	0.159	0.77 (0.38–1.57) *	0.724 ^#^
T/T-C/C	11	0.103	8	0.075	0.71 (0.27–1.83) *	0.722 ^#^
**Genotype**	**Control (*n* = 107)**		**Stroke (*n* = 107)**		**Crude OR (95% CI)**	***p***
	**Number**	**Frequency**	**Number**	**Frequency**		
**g.117803515C > T—*NOS1* (rs1879417) vs. c.-227G > C—*NOS2* (rs10459953)**						
C/C-C/C	0	0	6	0.056	-	-
C/C-C/G	1	0.009	13	0.121	14.66 (1.88–114.20) * ^B^ 43.67 (0.05–40,104.99)^0.070^ ^Cv^14.66 (1.88–114.20)	0.020 ^#^ 0.278 0.020 ^#^
C/C-G/G	0	0	9	0.0084	-	-
C/T-C/C	10	0.093	13	0.12	1.34 (0.56–3.21) *	0.759 ^#^
C/T-C/G	33	0.308	25	0.234	0.68 (0.37–1.26)	0.392 ^#^
C/T-G/G	31	0.290	11	0.103	0.28 (0.13–0.60) * ^B^ 0.53 (0.36–0.77)^0.935^ ^Cv^ 0.28 (0.13–0.60)	0.002 ^#^ 0.001 0.002 ^#^
T/T-C/C	8	0.075	10	0.093	1.28 (0.48–3.37) *	0.858 ^#^
T/T-C/G	11	0.103	13	0.121	1.21 (0.52–2.83) *	0.888 ^#^
T/T-G/G	13	0.121	7	0.065	0.51 (0.19–1.32) *	0.303 ^#^
**Genotype**	**Control (*n* = 107)**		**Stroke (*n* = 107)**		**Crude OR (95% CI)**	***p***
	**Number**	**Frequency**	**Number**	**Frequency**		
**g.117803515C > T—*NOS1* (rs1879417) vs. c.1823C > T (p.Ser608Leu)—*NOS2* (rs2297518)**						
C/C-C/C	0	0	19	0.178	-	-
C/C-C/T	0	0	8	0.075	-	-
C/C-T/T	1	0.009	1	0.009	1.00 (0.06–16.20) *	1.000 ^#^
C/T-C/C	7	0.065	34	0.318	6.65 (2.79–15.84) * ^B^ 2.66 (1.67–4.25)^0.472^ ^Cv^ 6.65 (2.79–15.84)	<0.001 ^#^ <0.001 <0.001 ^#^
C/T-C/T	21	0.196	15	0.140	0.67 (0.32–1.38) *	0.474 ^#^
C/T-T/T	46	0.430	0	0	-	-
T/T-C/C	0	0	28	0.262	-	-
T/T-C/T	11	0.103	1	0.009	0.08 (0.01–0.65) * ^B^ 0.03 (0.0001–22.48)^0.926^ ^Cv^0.08 (0.01–0.65)	0.036 ^#^ 0.289 0.036 ^#^
T/T-T/T	21	0.196	1	0.009	0.04 (0.01–0.29) * ^B^ 0.02 (0.0001–15.69)^0.987^ ^Cv^0.04 (0.01–0.29)	0.004 ^#^ 0.238 0.004 ^#^

* crude OR means OR calculated with conventional logistic regression; for the significant outcomes, the superscript ^B^ means the bootstrap-boosted OR (resampling with replacement, 1000 iterations); ^Cv^ means the cross-validated OR. Statistical power (1-β) for significant comparisons is given in superscripts. *p* < 0.05 along with the corresponding ORs are in red (for the combined genotypes increasing the risk of stroke) or in blue (for the combined genotypes with a protective effect); ^#^—denotes *p*-values with the Bonferroni correction.

**Table 9:** Distribution of haplotypes of the c.1823C > T (rs2297518) and c.-227G > C (rs10459953) polymorphisms in the *NOS2* gene and odds ratio (OR) with 95% confidence interval (95% CI) in patients with stroke and controls.

Haplotype	Control (*n* = 107)		Stroke (*n* = 107)		Crude OR (95% CI)	*p* *
	Number	Frequency	Number	Frequency		
TC	10	0.046	64	0.299	0.11 (0.06–0.23)	0.0001
TG	18	0.084	104	0.485	0.10 (0.06–0.17)	0.0001
CC	99	0.462	17	0.079	9.98 (5.68–17.53)	0.0001
CG	87	0.406	29	0.135	4.37 (2.71–7.04)	0.0001

*p* < 0.05 along with the corresponding ORs are in red (for the haplotypes increasing the risk of stroke) or in blue (for the haplotypes with a protective effect); *—denotes *p*-values with the Bonferroni correction.

## Data Availability

The data presented in this study are available on request from the corresponding author.

## Supplementary Materials

The following are available online at https://www.mdpi.com/2076-3425/11/3/391/s1, Table S1: Distribution of genotypes and alleles of the c.-89A > T (rs7943316) polymorphism in the *CAT* gene and odds ratios (ORs) with 95% confidence intervals (95% CIs) in patients with stroke and controls.
