# Supplementary material for: Single-Nucleotide Polymorphisms in Oxidative Stress-Related Genes and the Risk of a Stroke in a Polish Population—A Preliminary Study
Source: Brain Sci. 2021 Mar 19;11(3):391. doi: 10.3390/brainsci11030391 (PMC8003761; doi:10.3390/brainsci11030391)
Supplement: Supplementary file 1 [file brainsci-11-00391-s001.pdf]

Supplementary Table S1. Distribution of genotypes and alleles of the c.-89A>T (rs7943316) polymorphism in the *CAT* gene and odds ratios (ORs) with 95% confidence intervals (95% CIs) in patients with stroke and controls.

Genotype/Allele	Control (n = 107)		Stroke (n = 107)		Crude OR (95% CI)	*p*
	Number	Frequency	Number	Frequency		
A/A	20	0.187	15	0.140	0.71 (0.34-1.47)	0.357
A/T	46	0.430	59	0.551	1.63 (0.95-2.80)	0.076
T/T	41	0.383	33	0.308	0.72 (0.41-1.26)	0.251
χ^2 = 3.189; *p* = 0.2030						
A	86	0.402	89	0.416	1.06 (0.72-1.56)	0.766
T	128	0.598	125	0.584	0.94 (0.64-1.39)	0.766
